# A NEw MOdel of individualized and patient-centered follow-up for women with gynecological cancer (the NEMO study)—protocol and rationale of a randomized clinical trial

**DOI:** 10.1186/s13063-022-07022-0

**Published:** 2023-02-01

**Authors:** Stinne Holm Bergholdt, Dorte Gilsaa Hansen, Anna Thit Johnsen, Bo Snedker Boman, Pernille Tine Jensen

**Affiliations:** 1 Department of Clinical Medicine, Faculty of Health Science, University of Southern Denmark, Odense, Denmark; 2 Department of Gynecology and Obstetrics, Odense University Hospital, Odense, Denmark; 3 Center for Shared Decision Making, Lillebaelt Hospital, University Hospital of Southern Denmark, Odense, Denmark; 4 Institute of Regional Health Research, University of Southern Denmark, Odense, Denmark; 5 Research Unit of General Practice, Institute of Public Health, University of Southern Denmark, Odense, Denmark; 6 Department of Psychology, University of Southern Denmark, Odense, Denmark; 7 Department of Clinical Oncology and Palliative Care, Zealand University Hospital, Roskilde, Denmark; 8 Private practice, Hellerup, Denmark; 9 Department of Gynecology and Obstetrics, Aarhus University Hospital, Aarhus, Denmark; 10 Department of Clinical Medicine, Faculty of Health, Aarhus University, Aarhus, Denmark

**Keywords:** Cancer follow-up, Empowerment, Gynecological cancer, Quality of Life, Rehabilitation, Nurse intervention

## Abstract

**Background:**

Follow-up programs for gynecological cancer patients are currently under revision. There is limited evidence that traditional follow-up and clinical examinations improve survival in an early-stage gynecological setting. Further, traditional follow-up programs fail to accommodate the patient’s need for psychosocial and sexual supportive care and to actively involve patients and their relatives in the follow-up process. Individualized programs may replace traditional routine follow-up with fixed intervals and length. Focusing on alarm-symptoms and self-reporting may ensure detection of recurrence while allowing a continuous attention to the patient’s well-being and return to daily life.

In this study, a nurse-led, individualized, and need-based intervention with a specific focus on patient empowerment is tested against a standard physician-led model primarily focusing on the detection of recurrence.

**Methods:**

The study is designed as a clinical, randomized trial conducted in one of four national onco-gynecological centers in Denmark. Patients with early-stage cervical or endometrial cancer are eligible for inclusion. The intervention group undergoes individualized, nurse-led follow-up supporting patient empowerment including repeated use of electronic patient-reported outcome measures (ePROMs) before each contact as a dialogue support tool. The follow-up contacts are mainly conducted by telephone. All project nurses attended a special training program before project start and are all well-educated and dedicated onco-gynecological nurses. The control group receives standard, physician-led, follow-up without use of ePROMs or specific focus on empowerment.

The effect of the intervention is evaluated by questionnaires completed by patients at baseline (3 months after surgery) and 12, 18, and 36 months after surgery. Outcomes include empowerment using the Skill and technique subscale of the HEI-Q questionnaire as the primary outcome while fear of cancer recurrence and health-related quality of life as well as the remaining subscales of the HEI-Q represent secondary outcomes. Data is collected by use of the REDCap technology, which also provides a customized visual support function for the dialogue tool.

**Discussion:**

This study will provide new information about follow-up in early-stage gynecological cancer settings and thereby contribute to improvement of future follow-up programs. Importantly, the study will provide knowledge about the impact of specific focus on patient empowerment in follow-up programs and, further, how to facilitate empowerment among patients.

**Trial registration:**

The study is registered at clinicaltrials.gov: ID no. NCT03838861. Registered on 6 February 2019.

Protocol version 2, issue date 21 November 2022.

**Supplementary Information:**

The online version contains supplementary material available at 10.1186/s13063-022-07022-0.

## Introduction

Follow-up programs for gynecological cancer patients are currently under revision worldwide, and traditional routine follow-up, i.e., clinical examinations with fixed time intervals and length, is replaced by individualized programs in various forms and settings [1–3]. In this study, a nurse-led, individualized, and need-based intervention with a specific focus on patient empowerment is tested against a standard physician-led model primarily focusing on the detection of recurrence.

### Background and rationale

For decades, the purpose of follow-up after cancer treatment has been disease control and detection of recurrence only. However, in a low-stage gynecological cancer setting, recurrence rates are low and most often symptomatic, and there is limited evidence that follow-up including physical examinations improves survival [4–10]. Therefore, follow-up models enhancing patient education including information about alarm symptoms and easy access to clinical follow-up in case of symptoms or worry may replace the traditional programs. In addition, studies have shown that traditional follow-up programs fail to accommodate the patient’s need for psychosocial and sexual supportive care [11] and to actively involve patients and their relatives in the follow-up process [12]. Further, systematic guidance to self-manage symptoms and supportive care needs is requested by many patients but is often missing [13–15]. As a consequence, the latest Danish national as well as several international guidelines recommend that future follow-up programs are based on patient-centered and need-based approaches, thus encouraging support of patient empowerment. However, little evidence exists on how these follow-up programs are optimally organized.

The NEMO intervention is a new model of follow-up based on the following key elements:

- Systematic support of patient empowerment
- Nurse-led management of follow-up, based on telephone contacts
- Electronic patient-reported outcome measures (ePROMs) used as a dialogue support tool to support symptom evaluation and patient-provider communication

### Patient empowerment

Empowerment has been described as “a process by which people, organizations, and communities gain mastery over their affairs” [16] and empowerment processes as “empowering if it helps people to develop skills so that they can become independent problem-solvers and decision-makers” [17]. Being empowered includes three components: the intrapersonal component (individually perceived degree of control), the interactional component (understanding of the context of one’s options and choices), and the behavioral component (particular actions taken) [17]. The ability of self-management and self-care may therefore be considered as possible end-products of the process of patient empowerment.

Studies have shown that empowerment is of particular importance in follow-up and rehabilitation of cancer patients and improves patient outcomes when actively supported [18–21]. Ultimately, empowering patients to master the consequences of their cancer disease may improve quality of life [22]. Furthermore, it may reduce unmet needs and fear of cancer recurrence, because empowerment may improve coping mechanisms by increasing self-reflection and out-reaching for social and health-related support [18].

The impact of self-management and empowerment on patient outcomes in cancer follow-up has been investigated in various settings [15, 23, 24]. In a British study, patients with early-stage endometrial cancer agreed to replace 1–2 scheduled follow-up appointments with nurse-led consultations focusing on empowerment. High levels of satisfaction with the self-management contacts were observed, and the majority (71%) indicated a willingness to accept randomization in a future randomized controlled trial (RCT) comparing hospital follow-up with patient-initiated follow-up [15]. A systematic review of 42 studies of educational programs for cancer patients focusing on self-management indicated that self-management interventions reduce fatigue, pain, depression, anxiety, and emotional distress, and increase quality of life [24]. However, while these programs seem promising, due to inconsistent definitions, methodological problems, heterogeneity in interventions and outcomes, and lack of theoretical frameworks, a knowledge gap with respect to the science and corresponding translation of these interventions into practice still remains [23].

Supporting patient empowerment may promote patient involvement and reflections about symptoms and late effects, which may lead to more focused contacts to the health care system and increased health literacy in general. This process may further act to reduce social inequality, as the follow-up is tailored to the individual patient, taking into account her resources and challenges. On the other hand, a systematic focus on empowerment may cause a shift of responsibility towards the patients for the follow-up and general survivorship process, in which case socially disadvantaged patients’ risk being lost to follow-up. Therefore, a personal involvement of well-educated and engaged health care professionals is suggested to be crucial for the success of the follow-up course.

### Nurse-led, telephone-based follow-up

Different models of individually tailored and need based follow-up after cancer have been tested. Nurse-led models have been tested against physician-led, face-to-face models versus telephone or virtual contacts and other models focusing on improving self-management, empowerment, or self-care against traditional follow-up. Both nurse- and general practitioner-facilitated follow-up have shown equal survival and quality of life levels when compared to traditional follow-up and seems to provide a safe and stable environment [25, 26]. Specifically, nurse-led follow-up may facilitate coherency and compliance for vulnerable patients, optimize the involvement of both patients and relatives [27], and provide a holistic approach to unmet supportive care needs [28]. Furthermore, it has shown similar or improved patient satisfaction when compared to conventional physician-led follow-up [25].

Models based on digital contacts has been compared to usual face-to-face contacts in hospital outpatient settings. The video or telephone-based contacts have most often been carried out by specialist nurses [26, 28, 29]. Telephone-based follow-up has been described as a feasible [30], safe, and convenient high-quality alternative [31]. It provides patients with a sense of confidentiality and coherency and seems more efficient compared to face-to-face contacts [32, 33].

### Use of PROMs in follow-up

Various electronic patient-reported outcome measures (ePROMs) or PROMs (patient-reported outcome measures) have been developed and tested for the purpose of adequately addressing and monitoring cancer patients’ unmet needs. Additionally, PROMs are used for the planning of frequency, content, specialist level, etc., of follow-up consultations in order to further individualize the follow-up program [34, 35]. In a systematic review, use of PROMs was shown to improve patient-provider communication, treatment response, and patient satisfaction [34]. Further, in selected populations, the use of PROMS has demonstrated improvement in the quality of life, reduction in emergency room visits and hospitalizations, and improvement in quality-adjusted survival [36]. For those reasons, PROMs are suggested as important elements of future follow-up programs for cancer patients [34, 37]. However, PROM completion is not a neutral act of information retrieval but can change how patients think about their condition and follow-up [38]. How the clinician uses PROMs may be influenced by his/her relationship with the patient. Further, his/her professional role and boundaries may influence the patients’ experience of the follow-up process both negatively and positively [39].

### Hypothesis

A nurse-led follow-up program for cancer patients, including repeatedly and active use of ePROMs and a strong focus on providing patient empowerment, improves patient empowerment, patient involvement, and quality of life and reduce fear of cancer recurrence and the use of economic and human resources, when compared to a standard physician-led follow-up program.

## Methods

The study is designed as a clinical, parallel-group superiority trial with central randomization (1:1) conducted in one gynecological department. Patients with early-stage cervical or endometrial cancer are eligible for randomization. The intervention group undergoes an individualized, nurse-led follow-up program that supports patient empowerment and includes repeatedly use of ePROMs as a dialogue support tool.

The control group receives standard care, i.e., physician-led, individualized follow-up without use of ePROMs or focus on empowerment.

The study was developed and implemented—and results will be published—in accordance with the MRC Medical Research Council Guidelines on complex interventions [40] and the SPIRIT-PRO guideline [41] (Table 1).

**Table 1:** SPIRIT checklist for *Trials*

		**Reporting Item**	**Page and line number**	**Reason if not applicable**
**Administrative information**				
Title	#1	Descriptive title identifying the study design, population, interventions, and, if applicable, trial acronym	1 (1–2)	
Trial registration	#2a	Trial identifier and registry name. If not yet registered, name of intended registry	2 (61)	
Trial registration: data set	#2b	All items from the World Health Organization Trial Registration Data Set	3 (65)	
Protocol version	#3	Date and version identifier	2 (63)	
Funding	#4	Sources and types of financial, material, and other support	3 (65) + 14 (567–572)	
Roles and responsibilities: contributorship	#5a	Names, affiliations, and roles of protocol contributors	1 (5–21) + 14 (560–565)	
Roles and responsibilities: sponsor contact information	#5b	Name and contact information for the trial sponsor	3 (65)	
Roles and responsibilities: sponsor and funder	#5c	Role of study sponsor and funders, if any, in study design; collection, management, analysis, and interpretation of data; writing of the report; and the decision to submit the report for publication, including whether they will have ultimate authority over any of these activities	3 (65)	
Roles and responsibilities: committees	#5d	Composition, roles, and responsibilities of the coordinating center, steering committee, endpoint adjudication committee, data management team, and other individuals or groups overseeing the trial, if applicable (see Item 21a for data monitoring committee)	3 (65)	
**Introduction**				
Background and rationale	#6a	Description of research question and justification for undertaking the trial, including summary of relevant studies (published and unpublished) examining benefits and harms for each intervention	4 (67)–6 (166)	
Background and rationale: choice of comparators	#6b	Explanation for choice of comparators	4 (91)–6 (166)	
Objectives	#7	Specific objectives or hypotheses	6 (168–173)	
Trial design	#8	Description of trial design including type of trial (e.g., parallel group, crossover, factorial, single group), allocation ratio, and framework (e.g., superiority, equivalence, non-inferiority, exploratory)	6 (176–187)	
**Methods: participants, interventions, and outcomes**				
Study setting	#9	Description of study settings (e.g., community clinic, academic hospital) and list of countries where data will be collected. Reference to where list of study sites can be obtained	6 (189–196)	
Eligibility criteria	#10	Inclusion and exclusion criteria for participants. If applicable, eligibility criteria for study centers and individuals who will perform the interventions (e.g., surgeons, psychotherapists)	6 (207)–7 (217)	
Interventions: description	#11a	Interventions for each group with sufficient detail to allow replication, including how and when they will be administered	7 (252)–9 (304)	
Interventions: modifications	#11b	Criteria for discontinuing or modifying allocated interventions for a given trial participant (e.g., drug dose change in response to harms, participant request, or improving/worsening disease)	9 (305–310)	
Interventions: adherence	#11c	Strategies to improve adherence to intervention protocols, and any procedures for monitoring adherence (e.g., drug tablet return; laboratory tests)	13 (489–495)	
Interventions: concomitant care	#11d	Relevant concomitant care and interventions that are permitted or prohibited during the trial	9 (310)	
Outcomes	#12	Primary, secondary, and other outcomes, including the specific measurement variable (e.g., systolic blood pressure), analysis metric (e.g., change from baseline, final value, time to event), method of aggregation (e.g., median, proportion), and time point for each outcome. Explanation of the clinical relevance of chosen efficacy and harm outcomes is strongly recommended	10 (389)–11 (419)	
Participant timeline	#13	Time schedule of enrolment, interventions (including any run-ins and washouts), assessments, and visits for participants. A schematic diagram is highly recommended (see Figure)	7 (226–8 (258) + Fig. 2	
Sample size	#14	Estimated number of participants needed to achieve study objectives and how it was determined, including clinical and statistical assumptions supporting any sample size calculations	11 (421–432)	
Recruitment	#15	Strategies for achieving adequate participant enrolment to reach target sample size	7 (233–250)	
**Methods: assignment of interventions (for controlled trials)**				
Allocation: sequence generation	#16a	Method of generating the allocation sequence (e.g., computer-generated random numbers), and list of any factors for stratification. To reduce predictability of a random sequence, details of any planned restriction (e.g., blocking) should be provided in a separate document that is unavailable to those who enroll participants or assign interventions	7 (219–224)	
Allocation concealment mechanism	#16b	Mechanism of implementing the allocation sequence (e.g., central telephone; sequentially numbered, opaque, sealed envelopes), describing any steps to conceal the sequence until interventions are assigned	7 (219–224)	
Allocation: implementation	#16c	Who will generate the allocation sequence, who will enroll participants, and who will assign participants to interventions	7 (241–246)	
Blinding (masking)	#17a	Who will be blinded after assignment to interventions (e.g., trial participants, care providers, outcome assessors, data analysts), and how	7 (219–224)	
Blinding (masking): emergency unblinding	#17b	If blinded, circumstances under which unblinding is permissible, and procedure for revealing a participant’s allocated intervention during the trial	7 (222–224)	The design is open label with only outcome assessors being blinded so unblinding will not occur
**Methods: data collection, management, and analysis**				
Data collection plan	#18a	Plans for assessment and collection of outcome, baseline, and other trial data, including any related processes to promote data quality (e.g., duplicate measurements, training of assessors) and a description of study instruments (e.g., questionnaires, laboratory tests) along with their reliability and validity, if known. Reference to where data collection forms can be found, if not in the protocol	10 (389)–11 (419)	
Data collection plan: retention	#18b	Plans to promote participant retention and complete follow-up, including list of any outcome data to be collected for participants who discontinue or deviate from intervention protocols	10 (389)–11 (419)	
Data management	#19	Plans for data entry, coding, security, and storage, including any related processes to promote data quality (e.g., double data entry; range checks for data values). Reference to where details of data management procedures can be found, if not in the protocol	11 (434)–12 (443)	
Statistics: outcomes	#20a	Statistical methods for analyzing primary and secondary outcomes. Reference to where other details of the statistical analysis plan can be found, if not in the protocol	12 (445–467)	
Statistics: additional analyses	#20b	Methods for any additional analyses (e.g., subgroup and adjusted analyses)	12 (445–467)	
Statistics: analysis population and missing data	#20c	Definition of analysis population relating to protocol non-adherence (e.g., as randomized analysis), and any statistical methods to handle missing data (e.g., multiple imputation)	12 (445–467)	
**Methods: monitoring**				
Data monitoring: formal committee	#21a	Composition of data monitoring committee (DMC); summary of its role and reporting structure; statement of whether it is independent from the sponsor and competing interests; and reference to where further details about its charter can be found, if not in the protocol. Alternatively, an explanation of why a DMC is not needed	N/A	A DMC has not been established. Data will be anonymized and stored according to all applicable guidelines regarding data management and analyses and data protection act. Data management and analyses are independent from sponsors and funders, and none of the authors have any conflicts of interest
Data monitoring: interim analysis	#21b	Description of any interim analyses and stopping guidelines, including who will have access to these interim results and make the final decision to terminate the trial	N/A	Since no serious harms was expected, no plan has been made for interim analyses
Harms	#22	Plans for collecting, assessing, reporting, and managing solicited and spontaneously reported adverse events and other unintended effects of trial interventions or trial conduct	12 (469–486)	
Auditing	#23	Frequency and procedures for auditing trial conduct, if any, and whether the process will be independent from investigators and the sponsor	13 (489–495)	
**Ethics and dissemination**				
Research ethics approval	#24	Plans for seeking research ethics committee/institutional review board (REC/IRB) approval	14 (548–555)	
Protocol amendments	#25	Plans for communicating important protocol modifications (e.g., changes to eligibility criteria, outcomes, analyses) to relevant parties (e.g., investigators, REC/IRBs, trial participants, trial registries, journals, regulators)	14 (559–562)	
Consent or assent	#26a	Who will obtain informed consent or assent from potential trial participants or authorized surrogates, and how (see Item 32)	7 (241–246) + 14 (545–549)	
Consent or assent: ancillary studies	#26b	Additional consent provisions for collection and use of participant data and biological specimens in ancillary studies, if applicable	N/A	No ancillary studies are planned, and no biological specimen were collected
Confidentiality	#27	How personal information about potential and enrolled participants will be collected, shared, and maintained in order to protect confidentiality before, during, and after the trial	11 (434–439)	
Declaration of interests	#28	Financial and other competing interests for principal investigators for the overall trial and each study site	15 (570–577)	
Data access	#29	Statement of who will have access to the final trial dataset, and disclosure of contractual agreements that limit such access for investigators	15 (593–596)	
Ancillary and post trial care	#30	Provisions, if any, for ancillary and post-trial care, and for compensation to those who suffer harm from trial participation	15 (598–601)	
Dissemination policy: trial results	#31a	Plans for investigators and sponsor to communicate trial results to participants, healthcare professionals, the public, and other relevant groups (e.g., via publication, reporting in results databases, or other data sharing arrangements), including any publication restrictions	15 (604–611)	
Dissemination policy: authorship	#31b	Authorship eligibility guidelines and any intended use of professional writers	15 (610–611)	
Dissemination policy: reproducible research	#31c	Plans, if any, for granting public access to the full protocol, participant-level dataset, and statistical code	15 (609)	
**Appendices**				
Informed consent materials	#32	Model consent form and other related documentation given to participants and authorized surrogates	Additional file 1: Appendix 1 and Additional file 2: Appendix 2	
Biological specimens	#33	Plans for collection, laboratory evaluation, and storage of biological specimens for genetic or molecular analysis in the current trial and for future use in ancillary studies, if applicable	N/A	No biological specimen was collected

It is strongly recommended that this checklist be read in conjunction with the SPIRIT 2013 Explanation & Elaboration for important clarification on the items. Amendments to the protocol should be tracked and dated. The SPIRIT checklist is copyrighted by the SPIRIT Group under the Creative Commons “Attribution-NonCommercial-NoDerivs 3.0 Unported” license. This checklist can be completed online using https://www.goodreports.org/, a tool made by the EQUATOR Network in collaboration with Penelope.ai

### Setting

The study was initiated at the Department of Gynecology, Odense University Hospital (OUH), Denmark. The department is one of four national onco-gynecological centers and yearly treat around 400–450 patients with gynecological cancer and borderline disease, of which 70–80 have cervical cancer and 150–160 have endometrial cancer. Overall, about 17% of the patients with cervical cancer, and 60% of patients with endometrial cancer are expected to meet the inclusion criteria (numbers based on the 2016/2017 annual report from DGCD (Danish Gynecological Cancer Database) at dgcd.dk).

### Patient and public involvement

During the study design phase, 10 patient representatives previously treated for cervical or endometrial cancer at the department took part in a 1:1 discussion with SHB or a project nurse about the intervention, the proposed recruitment procedures, the outcomes, and all patient materials including questionnaires and symptom lists used to support patient-provider communication. Revisions were made in accordance. In addition, patient representatives and relatives will be asked to participate in the interpretation and dissemination of the study results.

### Eligibility criteria

**Fig. 1:**
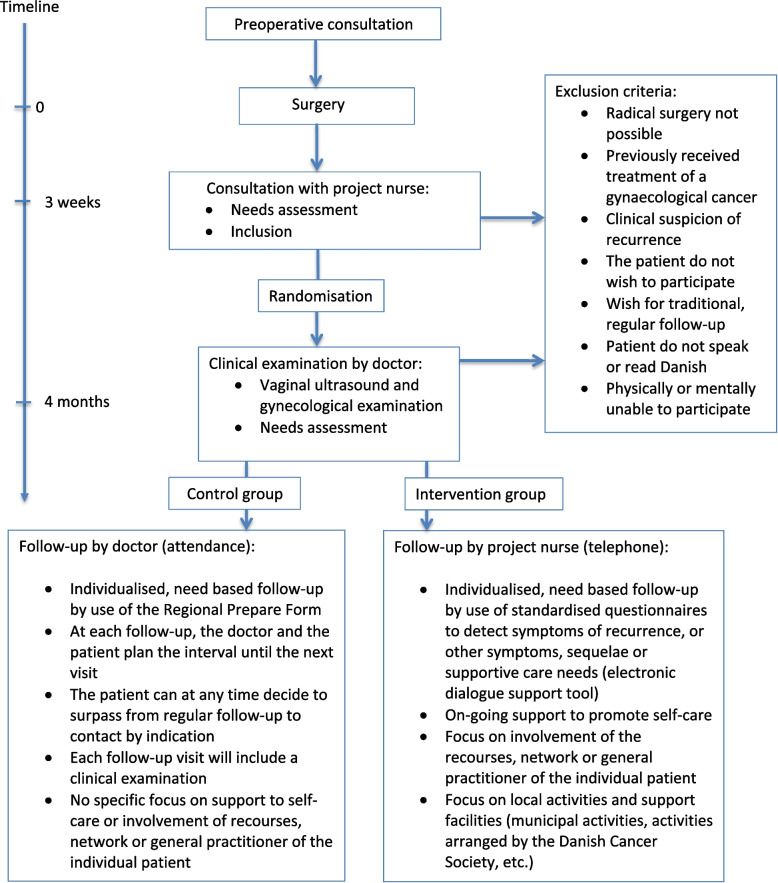
Flowchart

All adult women with confirmed early-stage cervical cancer (FIGO stage 1A1–1A2 treated with cone biopsy, loop electrosurgical excision procedure or simple hysterectomy AND no recommendation for sentinel node mapping (tumor < 7 mm)) or low-stage endometrial cancer (FIGO stage 1 endometrioid adenocarcinoma grade 1 and 2 without lymphoid vascular invasion) were invited to participate. All patients were surgically treated at the Department of Gynecology, OUH, were not candidates for oncological treatment pre- or postoperatively, were able to read and speak Danish, and physically and mentally able to participate (Fig. 1).

Patients, who did not wish to participate received standard physician-led, need based follow-up, which was implemented as the standard follow-up program during the autumn of 2017.

### Randomization and blinding

The allocation sequence of the randomization is computer-generated with varying block sizes. Block sizes are kept unknown for all investigators. Due to the study design, patients and clinicians involved in the treatment are informed about allocation status. During data processing and statistical analyses, the statistician and the research group will be blinded.

### Participant timeline, recruitment, and allocation

From March 2019, patients were consecutively invited to participate in the study at the prescheduled encounter 3–4 weeks after primary surgery.

**Fig. 2:**
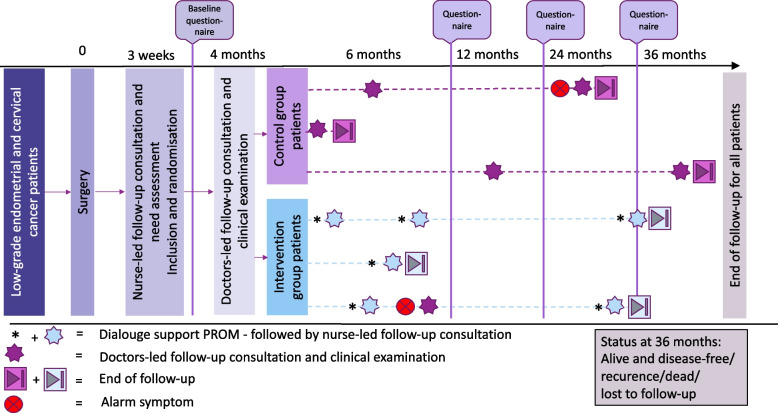
Timeline; 6 examples

All patients (i.e., patients in the intervention group and in the standard treatment group) follow the same follow-up trajectory during the first four months after surgery (Fig. 2), which include:

1. *A nurse-led consultation 3–4 weeks* after primary surgery, including in-consult needs assessment led by the nurse using a one-paged tick-off list of the most common physical and psychosocial problems after cancer treatment. Further, symptoms and other post-operative sequelae are reviewed using a disease specific list of alarm symptoms prepared by working groups of the Danish Gynaecological Cancer Group (DGCG) [42], and the patient is carefully instructed to be aware of and contact the department in case of symptoms from the list as they may indicate recurrence. Patients are reassured that access to clinical examination is available and provided when needed during the entire follow-up period (up to 3 years after primary surgery).
  At this time, disease stage and indication for adjuvant therapy has been clarified, and patients who meet the inclusion criteria will be given oral and written information about the project by the project nurse. Participants give their written consent. Next, the nurse creates a case report in the REDCap (Research Electronic Data Capture) system and enter basic information about the patient. A randomization button will appear, and the randomization is carried out using the Randomization Module within REDCap. The patients are informed about their randomization status by the nurse.
  In the rare case a patient fails to attend this consultation, the nurse will call the patient and make another appointment. This procedure ensures that most eligible patients will be given the opportunity to receive information about the study.
2. *A follow-up consultation with a physician 4 months postoperatively*, where a clinical assessment is performed, and ‘the preparatory form’ and ‘symptom list’ are reviewed as part of the need assessment. In case the symptom report or the clinical examination indicate potential persistent disease, the patient is excluded from the study. Otherwise, the next encounter in their follow-up is planned in accordance with their allocation status and needs. The patients in the intervention group are scheduled for a nurse-led consultation, most often by phone, and patients in the control group are scheduled for a physician-led consultation at the outpatient clinic.

#### The control group

The physician-led usual care program consists of out-patient face-to-face consultations including a gynecological examination and a transvaginal ultrasound examination and scheduled based on individualized need assessment. At the end of each consultation, the patient and the physician agree on if or when to schedule a new consultation based on the needs of the patient and guided by disease-related needs, sequelae after surgery, compliance, comorbidity, and other relevant factors.

#### The intervention group

The nurse-led model is led by a project nurse and based on phone contacts with content and intervals individually planned in agreement with the patient. In some cases, attendance in the outpatient clinic may be preferred by the patient or by the project nurse, i.e., in case of complex supportive care needs or when active involvement of a relative is considered essential for the empowerment process.

During encounters, the nurse will be supported by graphic visualization of the results of the ePROMs completed in REDCap by the patient before each contact. The ePROMs include a disease specific symptom list and the generic European Organization for Research and Treatment of Cancer Quality of Life Questionnaire (EORTC QLQ-C30) [43] supplemented with the relevant disease-specific add-on questionnaire modules (EORTC EN24 or EORTC CX24) [44, 45]. Thresholds for the scores are pre-defined and visualized to health professionals with colors to indicate severe problems (red), no problems (green) and mild or moderate problems (yellow and orange), respectively. Hence, alarm symptoms and problem areas are clearly presented to the nurses and changes over time can be visualized thus improving the interpretation. This feature of the REDCap is comparable to that of the AmbuFlex technology [46], which has been used for similar projects in lung, prostate, breast, and gynecological cancer settings [47–50].

Each consultation includes:

1. Review of the PROM in collaboration between clinician and patient with specific attention to alarm symptoms
2. Dialogue facilitating identification and utilization of available resources (own, relatives, general practitioner, supportive care activities in the local municipality, and in the local community in general)
3. Support of patient empowerment and self-management
4. Referral to relevant support if needed
5. Referral to medical examination if needed
6. Shared decision of time and target of future follow-up

At the end of each consultation, the patient and the nurse agree on if and when to schedule a new consultation.

#### Duration of the programs

Both follow-up programs have a maximum duration of 3 years, regardless of allocation status. All patients in both groups may at any time request to end their formal follow-up. In this case, no new contact is planned, but the patient can contact the department directly in case of symptoms or worry for up to 3 years after surgery.

#### Modifications or discontinuation of the intervention

If a patient in the intervention group wish to withdraw their consent or in the rare case of recurrence, the patient will be evaluated by a doctor and a plan for future follow-up or further investigations will be made. There are no restrictions regarding concomitant care during the trial.

### Training of the project nurses

A total of six project nurses were trained to lead the intervention group consultations. The training program was based on theories about professional learning (knowledge, role-play and supervision may enable professionals to change clinical behavior), health communication, the bio-psycho-social understanding of diseases like cancer, and holistic needs assessment (includes the variety of problems and needs that may follow cancer treatment).

The training program included a pre-study 2-day seminar and brush-up training during the study period. The project nurses were all experienced within the onco-gynecological field from years of engagement in the department and had applied for the position as project nurse specifically on this project.

The training at the 2-day seminar was led by chief psychologist BSB who is specialized in rehabilitation and empowerment of cancer patients. Experienced medical physicians and psychologists provided a mixture of interactive lectures and role plays with a professional actor as the patient. The theoretical themes were empowerment, adjustment psychology, and benefits and risks when using PROMs as dialogue support tool. Further, the educational program included medical aspects of endometrial and cervical cancer with special attention to alarm symptoms, recurrence, late effects and normal physical, social and psycho-sexual reactions, and problems related to gynecologic cancer. The clinical cases used for role play were described as difficult situations by the project nurses or by the study physicians (PTJ and SHB) beforehand and were reviewed with psychological aspects by the course leader.

Brush-up training is planned annually during the inclusion period and will be supervised by SHB and BSB.

### Theoretical frameworks for the intervention

This nurse-led intervention and the teaching program to nurses were designed based on theories about (1) empowerment as an important component in enabling people in handling (nurses are empowered and patients are empowered), (2) attachment as a way to understand the individual patient ‘s behavior and trust in clinical consultations and professional relationships, and (3) PROM in the clinical consultation as a relevant dialogue tool (conceptualizes to patients and professionals what may be relevant issues and serves as a method to target delivery of supportive care in line with the patient’s needs).

**Fig. 3:**
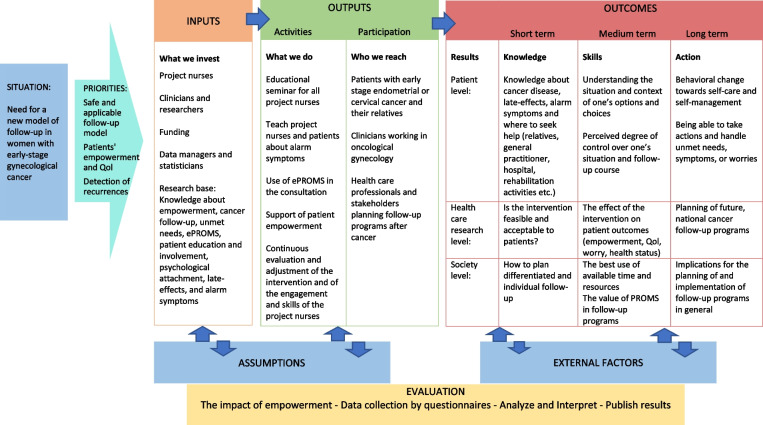
The logic model of the intervention

The theoretical frame of the intervention is summarized in the logic model (Fig. 3). The logic model visualizes the assumptions and theory of action that underlie the structure of the intervention, and it defines the four components of the models: resources, activities, outputs, and outcomes, and explains how they connect.

1. Theoretical framework of empowerment
  A central part of the program was to support the empowerment of patients in a relational context. Lately, more attention has been given to how to encourage the patients to handle their own supportive care process, i.e., how to optimize the empowering processes supported by the health care providers [51]. It is acknowledged that the health care providers play an important role in encouraging, accepting and enabling the patient to be empowered [51], for example, by providing adequate information to the patients on how to navigate. However, it is important to note that there is not a direct link between the enabling of empowerment and empowerment outcomes. Thus, the empowerment of the patient is a process pending on the relationship between the patient and the health care provider but also involves the patient’s relatives, her network, her general practitioner, local supportive care activities, and her motivation for being empowered.
2. Theoretical framework of attachment in the clinical context
  Understanding the patient’s premises of trusting clinicians is crucially important for the clinician’s ability to support the patient during cancer follow-up. Attachment theory explains differences in close relationships under threatening circumstances, as in this case, a cancer diagnosis [52]. By applying this theory, often held assumptions such as “everybody wants information” or “it is always good to talk about emotions” were discussed and nuanced. In accordance, theory about attachment styles and how to understand different attachment orientations during clinical encounters was part of the educational training of the project nurses.
3. Theoretical framework of PROM as a dialogue supportive tool

Using PROM as a supporting dialogue tool in clinical consultations is based on the assumptions that (1) filling in a questionnaire regarding potential problems and needs that may occur during or after cancer treatment prompts a process of self-reflection and supports the patient to raise these issues with the clinician, (2) involving PROM responses into the consultation raises the clinician’s awareness and pave the way for setting the agenda to discuss and deal with the patient’s worries and concerns, and (3) by effectively detecting needs in a shared dialogue, a crucial step is taken towards being professionally advised and supported [39]. Visual summaries automatically produced by cut-off values to show areas of concern may facilitate their communication, by easily illustrating actual as well as previous problem levels.

However, some concerns concerning the impact of PROM on referral and the patient’s well-being is relevant to consider [34, 39, 53–58]. For instance, standardized checklists and frameworks can narrow discussion and disrupt the process of managing and building relationships with patients. Further, clinicians across a range of clinical settings found that using a standardized PROM during initial assessments could constrain, rather than support communication and interfered with the process of managing relationships with patients [39]. Our intervention and learning programs sought to address these obstacles.

### Outcomes

**Fig. 4:**
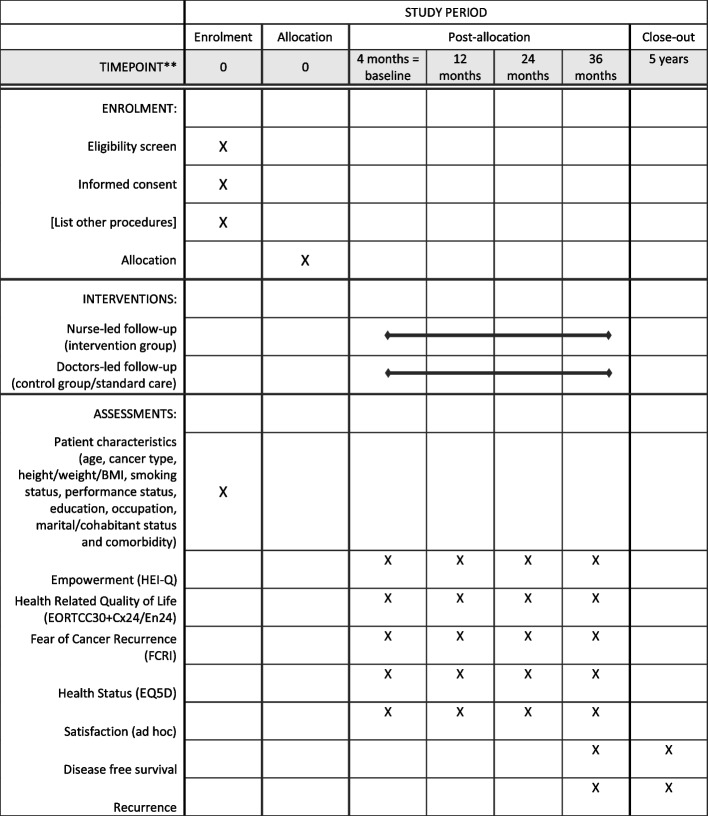
SPIRIT schedule of enrolment, interventions, and assessments

The effect of the intervention is evaluated by questionnaires administered to all patients at baseline (3 months postoperatively) and 12, 18, and 36 months postoperatively. The questionnaires assess empowerment, fear of cancer recurrence, and health-related quality of life (Fig. 4).

The primary outcome is change in patient empowerment from baseline to 12 months as measured by the Skill and technique subscale of the validated Health Education Impact Questionnaire (HEI-Q) [59, 60]. The HEI-Q was developed to assess the effects of health education programs on self-management among individuals with chronic conditions, including cancer [59], and has been validated in Danish [61]. The 40-item questionnaire comprises five subscales each conceptualized as key aspects of patient empowerment, including social integration and support, health service navigation, constructive attitudes and approaches, skill and technique acquisition, and emotional distress. The skill and technique acquisition construct aims to capture the knowledge-based skills and techniques that persons acquire (or re-learn) to help them cope with symptoms and health problems, which was found essential in order to evaluate the intervention, and therefore chosen as the primary outcome.

The secondary outcomes include fear of cancer recurrence (Fear of Cancer Recurrence Inventory, FCRI) [62], patient involvement (CollaboRATE) [63], health status (EQ5D) [64–66], and Quality Of Life (EORTC QLQ C30) [43] including disease-specific late effects using the QLQ-CX24 module [44] for cervical cancer and the QLQ-EN24 [45] for endometrial cancer. The baseline questionnaire furthermore obtained information on socio-demographic characteristics: education, employment status, income, cohabitant status, comorbidity, height/weight (BMI), and smoking status.

Data from the National Patient Register and the Danish Pathology Register regarding disease-free survival (DFS) and disease recurrence (IDR) will be obtained for all patients after 3 and 5 years of follow-up.

Further, the number, type, and duration of all contacts to the department in the two groups are registered and, in combination with the five questions about health status of the EQ5D questionnaire [66], used to calculate QALYs at a health economic evaluation of cost-effectiveness 5 years postoperatively.

### Sample size

Based on previous studies, we expect the standard deviations (SD) of the five scales in the HeiQ Empowerment questionnaire to be around 0.43 [59]. A conservative estimate of the standard deviation of the mean change between two time points is given by multiplying this SD with the square root of 2. A difference between 5 and 10 on a 0 to 100 scale is often judged to be a clinically relevant difference [67]. Therefore, we wish to be able to detect a treatment effect of 0.21 points on the Skill and technique acquisition-scale of the HeiQ (corresponding to a 7-point difference on at 0–100 scale) from baseline to 12 months follow-up. With a risk of type I error of 0.05 and type II error of 0.20 (power 80%), we need 268 patients (randomized 1:1 in each treatment arm) to complete the trial. As we expect an attrition of 30% at 12 months follow-up including death, withdrawal, and non-participation in the follow-up questionnaires, we aim to randomize 384 patients (192 in each arm).

### Data collection methods and management

Data storage and handling of the ePROM are managed by the Open Patient data Explorative Network (OPEN), a non-profit research infrastructure of OUH and University of Southern Denmark. OPEN uses the REDCap technology and provide secure (anonymized) data storage and analysis environments. Further, OPEN provides data management assistance and statistical support.

Reminder procedures were incorporated in the automated electronic transmission of questionnaires, and two reminders were sent after 1 and 2 weeks, respectively. At the end of follow-up (3 years from baseline), number, type (nurse versus physician-led), and dates of follow-up of each patient were retrieved from the electronic patient files.

### Statistical methods

Descriptive statistics will be used to analyze participation rates and possible (random) group differences regarding baseline socio-demographic and clinical characteristics and non-participation at follow-up.

Missing data and scores based on the FCRI and EORTC scales will be handled and reported in accordance with guidelines [62, 67–69].

Linear mixed effects models, modeling timepoint as well as interaction between time points and intervention group as fixed effects and random effects on patient level, will be used for the analyses. The models will not include a fixed effects term for treatment group; thus, a common baseline measurement of the treatment groups will be induced, which is in accordance with suggested methods in the literature [70]. Model assumptions will be tested using visual inspection of qq-plots of residuals as well as residuals versus fit plots. If the model assumptions are not met, bootstrapping procedures (in case of non-normally distributed residuals) and/or appropriate modifications of the estimated variance–covariance matrix (in case of violations of variance-homogeneity assumption) will be included in the analyses.

For each outcome and at each time point (3, 6, and 9 months of follow-up), we will compare mean differences from baseline to follow-up for the intervention and control group and apply relevant regression analysis with adjustment of age and disease type. Also, subgroup analysis for disease and age specific groups will be applied.

A significance level of 0.05 is applied, and all analysis will be conducted using the STATA software.

### Harms

The participants all have early-stage disease with a very low risk of recurrence (8.5% of the endometrial cancer patients with no lymph-vascular space invasion [8] and 1.6% of stage 1A1–1A2 cervical cancer patients [9]). In the control group, patients are treated “as usual,” and in the intervention group, patients are repeatedly informed about alarm symptoms and how to react adequately. Furthermore, they are asked to report signs and symptoms with regular intervals by using the ePROMs and the PROMs are viewed and interpreted by the project nurses.

If the project nurses are in doubt about how to handle a patient or situation, they can always contact SHB or another physician in the onco-gynecologic team to arrange a clinical encounter and examination within a few days. The specific information and repeated focus on alarm symptoms may therefore ensure timely action in case of symptoms but may on the other hand introduce or increase levels of fear of recurrence. Levels of fear of recurrence is measured in both groups and is addressed by the nurse when relevant.

Summing up, we assume that the risk of overlooking recurrent disease is minimal, and the risk of harm in this regard is accordingly small. However, cases of recurrence in both groups will be investigated to determine whether the type of follow-up had any influence on a possible delay of the diagnosis or other potential negative impact for the patient.

### Auditing

To secure protocol fidelity, a half-day seminar was held 6 months after start of inclusion. Key points from the training seminar were repeated, and difficult patient cases, understanding of the PROMs and their use during clinical encounters, and logistical issues were discussed. As previously described, similar seminars are run annually during the inclusion period. Further, shorter meetings addressing protocol affiliation issues is scheduled every 6 months.

## Discussion

This study will provide new information about follow-up in early-stage gynecological cancer setting and thereby contribute to improvement of future follow-up programs. Importantly, the study will provide knowledge about the impact of a specific focus on patient empowerment in follow-up programs and, further, how to facilitate empowerment to the patients.

Due to the study design, there are some limitations and risk of bias. First, patient inclusion is dependent of the information and recruitment ability of the project nurse, which may be affected by the personal chemistry between patient and nurse. Also, those patients who are most afraid of recurrence may not wish to participate. Thus, there may be a selection bias that may affect the generalizability of results. This will be discussed in the main paper. Similarly, the effect of the intervention on patient level may reflect the personal relationship between patient and nurse and by the involvement and commitment by each individual nurse to the project. However, as the study includes several different nurses, we believe the intervention to reflect what may be realistically expected from the intervention. Further, study outcomes are primarily based on patient questionnaires, which are possible subject to bias due to selection and recall bias. However, most of the possible bias will be the same in both arms and should therefore not affect the results. It is however possible that patients in the intervention arm wish to express their gratitude by giving more positive feedback.

Finally, the intervention is not designed or dimensioned to statistically detect a difference in cancer recurrence between the two groups. Cases of recurrence will be assessed for all patients after 3 and 5 years of follow-up and potential differences in the two groups reported. However, due to the low number of expected recurrences and the aim of the trial, this is not the primary outcome of the trial.

Results from this study may implicate revisions and improvements in the organization of follow-up in other early-stage cancer settings, where recurrence rates are low, and self-management and self-referral is the most important factor of the follow-up process.

## Trial status

Patient inclusion started on 1 March 2019 and is expected completed by 31 December 2022.

## Supplementary Information

**Additional file 1. Appendix 1: **Patient information NEMO (in Danish).

**Additional file 2. Appendix 2: **Patient consent form NEMO (in Danish).

## Abbreviations

NEMO: NEw MOdel of follow-up
PROMs: Patient-reported outcome measures
ePROMs: Electronic patient-reported outcome measures
OUH: Odense University Hospital
DGCD: Danish Gynecological Cancer Database
FIGO: International Federation of Gynecology and Obstetrics
EORTC QLQ-C30: European Organization for Research and Treatment of Cancer Quality of Life Questionnaire
HEI-Q: Health Education Impact Questionnaire
FCRI: Fear of Cancer Recurrence Inventory
EQ5D: European Quality of life 5 Domain (Health Status Questionnaire)
REDCap: Research Electronic Data Capture
OPEN: Open Patient data Explorative Network
MDT: Multidisciplinary team
