# Supplementary material for: A NEw MOdel of individualized and patient-centered follow-up for women with gynecological cancer (the NEMO study)—protocol and rationale of a randomized clinical trial
Source: Trials. 2023 Feb 1;24:74. doi: 10.1186/s13063-022-07022-0 (PMC9890753; doi:10.1186/s13063-022-07022-0)
Supplement: Supplementary file 1 — Additional file 1. Appendix 1: Patient information NEMO (in Danish). [file 13063_2022_7022_MOESM1_ESM.doc]

**Deltagerinformation
- om deltagelse i en videnskabelig undersøgelse**

**Fra kontrol til opfølgning**

**efter kræft i underlivet**

**
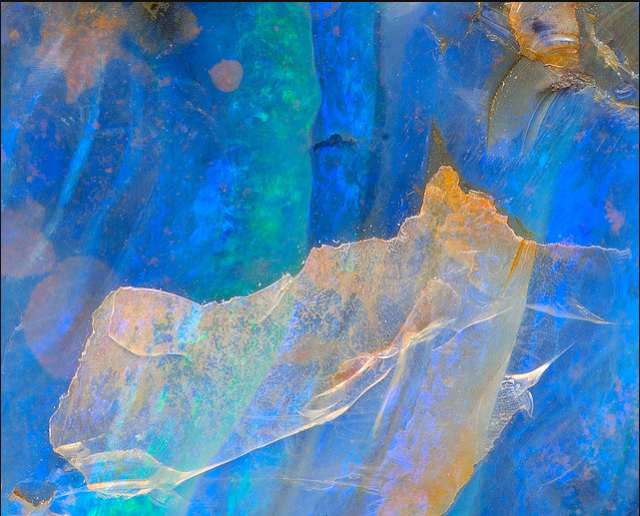
**

**Gynækologisk Obstetrisk Afdeling**

**ENDOmetriecancerpatienters KONtrolforløb – en RCT**


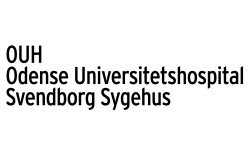


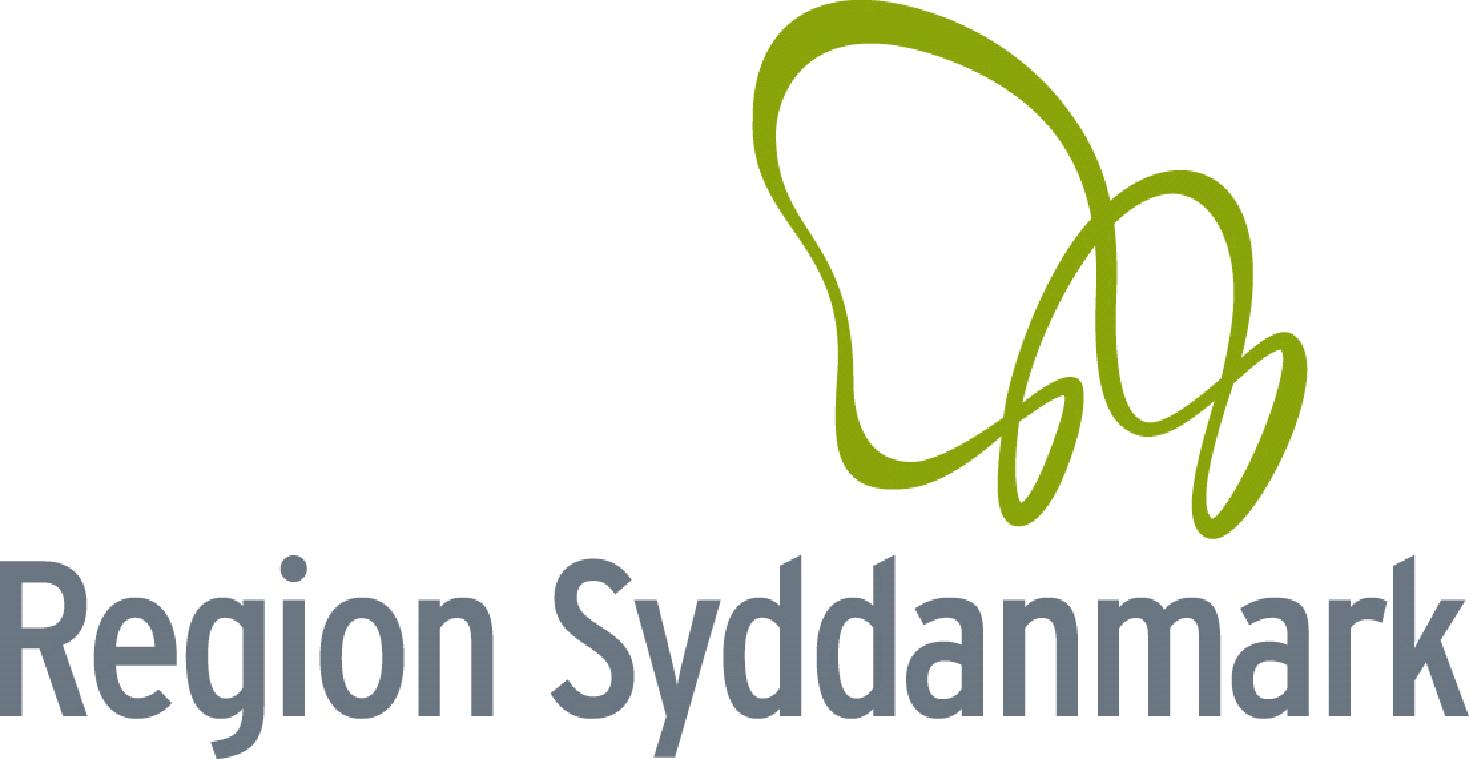


Du forespørges hermed, om du vil deltage i det sundhedsvidenskabelige forskningsprojekt:

***Fra kontrol til individualiseret opfølgning for kvinder med gynækologisk kræft – et randomiseret, kontrolleret studie***

Før du beslutter, om du vil deltage i undersøgelsen, er det vigtigt, at du forstår, hvad undersøgelsen går ud på, og hvorfor den gennemføres. Vi vil derfor bede dig om at læse denne deltagerinformation grundigt.

BAGGRUND

I 2015 besluttede Sundhedsstyrelsen, at de traditionelle kontrolforløb efter afsluttet behandling for kræft i underlivet, skal erstattes med et behovsorienteret opfølgningstilbud, der planlægges i samarbejde med patienten. Denne opfølgning kan varetages af såvel læger som sygeplejersker, ligesom det anbefales, at den praktiserende læge og kommune inddrages i et tæt samarbejde om den enkelte patients opfølgningsforløb. Endvidere lægges stor vægt på at støtte og styrke patientens evne til at drage omsorg for sig selv, samt at instruere patienten i at henvende sig ved symptomer på tilbagefald.

Det nye opfølgningsprogram er baseret på store undersøgelser der viser, at kontrolforløb med faste intervaller ikke øger overlevelsen, men derimod kan fastholde patienterne i en unødig sygerolle og i nogle tilfælde give falsk tryghed.

FORMÅL

Formålet med dette projekt er at teste to forskellige former for behovsorienteret opfølgning efter kirurgisk behandling af kræft i underlivet.

DE TO TYPER OPFØLGNING

*Patienter, der skal følges i en lægestyret gruppe*, aftaler fra gang til gang med lægen, hvornår de skal ses igen, og bliver ved hvert besøg tilbudt en gynækologisk undersøgelse, ultralydsscanning, samt en kort samtale om aktuelle symptomer og problemer. Besøgene varer typisk ca. 10-15 min.

*Patienter, der skal følges i den sygeplejerskestyrede gruppe*, aftaler ligeledes med sygeplejersken fra gang til gang, hvornår den næste kontakt skal foretages. Kontakterne er overvejende telefoniske, hvor typiske symptomer og problemer efter behandling for kræft i underlivet gennemgås, og hvor der hver gang vil blive spurgt grundigt ind til eventuelle symptomer på tilbagefald. Såfremt der skulle være tegn til dette, bookes hurtigst muligt tid til at blive set i ambulatoriet af en læge. Derudover er der mulighed for kontakt med fremmøde, hvis der skulle være særligt brug for dette. Forud for hver kontakt udfylder patienten et kort, elektronisk spørgeskema, som sygeplejersken læser igennem før kontakten. Samtalen kan dermed målrettes de emner eller områder, hvor der opleves problemer. Sygeplejersken kan derudover se udviklingen i symptomer og problemer fra gang til gang, og har dermed et godt overblik over sygehistorien, hvordan den ændrer sig over tid, og hvilken type af støtte der er brug for.

I denne gruppe vil der være særligt fokus på at styrke patienternes egenomsorgsevne, dvs. evnen til selv at reagere og håndtere problemer og udfordringer efter sygdommen, og til bedre at udnytte de ressourcer man selv har, og som man har adgang til i sit netværk og omgivelser.

Kontakterne er som udgangspunkt ikke tidsbegrænsede, og kan tage den tid, der er brug for.

Alle patienter (i begge grupper) kan til enhver tid i opfølgningsforløbet, fx ved akutte problemer, uforklarlige symptomer eller utryghed, kontakte afdelingens forløbskoordinator og bede om at blive set af eller tale med en sygeplejerske eller den patientansvarlige læge.

For begge grupper gælder det, at opfølgningsforløbet afsluttes efter senest 3 år.

PLAN FOR PROJEKTET

Projektet gennemføres på gynækologisk afdeling, Odense Universitetshospital, og patienter inkluderes i perioden fra 1. marts 2019 og frem til medio 2022. Patienter, der har givet tilsagn om deltagelse, fordeles ved lodtrækning, dvs helt tilfældigt, til et behovsorienteret opfølgningstilbud ved en læge (kontrolgruppe) eller ved en sygeplejerske (interventionsgruppe). Fordelingen sker i forbindelse med opfølgningsbesøget 4 uger efter operationen. Alle patienter ses derefter til en gynækologisk undersøgelse og indvendig scanning 4 måneder efter operationen. Herefter aftales det videre forløb med enten lægen eller sygeplejersken, der skal varetage opfølgningsbesøget, afhængigt af, hvilken gruppe man blev fordelt til ved lodtrækningen.

Patienter i begge grupper tilsendes elektroniske spørgeskemaer, der skal udfyldes i alt 5 gange, første gang 4 uger efter operationen, og derefter hhv. 4, 12, 24 og 36 måneder efter operationen.

Spørgeskemaerne vil bl.a. indeholde spørgsmål om dine udfordringer, symptomer og livskvalitet efter sygdommen, samt om oplysninger som uddannelse, arbejde, tobaksforbrug, dit forbrug af medicin, besøg hos egen læge og brug af andre sundhedsydelser, som fx genindlæggelse på hospitalet eller konstatering af tilbagefald. Vi vil også bede om tilladelse til at slå helbredsoplysninger op i din journal.

Alle data vil blive opbevaret i anonymiseret form, og offentliggørelsen vil blive gjort på en måde, så ingen resultater er personhenførbare.

Der er ingen forventede bivirkninger, gener eller risici ved deltagelse i undersøgelsen.

ØKONOMISK STØTTE

Projektet har opnået økonomisk støtte på 700.000 kr. fra Region Syddanmark, der udelukkende anvendes til aflønning af den projektansvarlige læge. Derudover er der bevilget 700.000 kr. fra Kræftens Bekæmpelse til bl.a. aflønning af projektsygeplejerskerne samt 400.000 kr. fra Odense Universitetshospital til bl.a. sygeplejerskernes uddannelsesforløb.

Projektet er anmeldt til Datatilsynet og er godkendt af Den Regionale Videnskabsetiske Komité.

NYTTE VED UNDERSØGELSEN
Din deltagelse kan få stor betydning for, hvordan opfølgningen af kvinder med kræft i underlivet kommer til at foregå i fremtiden.

Vi håber, at du med denne information har fået tilstrækkeligt indblik i, hvad det vil sige at deltage i undersøgelsen, og at du føler dig rustet til at tage beslutningen om din eventuelle deltagelse. Det er frivilligt at deltage i forsøget. Du kan når som helst og uden at give en grund trække dit samtykke tilbage uden det får konsekvenser for din videre behandling.

For yderligere information om projektet kan den projektansvarlige læge kontaktes. For yderligere information om forsøgspersoners generelle rettigheder, se venligst vedlagte tillæg fra Videnskabsetisk Komité.

*Projektansvarlig:*

Afdelingslæge Stinne Holm Bergholdt

[stinne.holm.bergholdt@rsyd.dk](mailto:stinne.holm.bergholdt@rsyd.dk)

Tlf: 40 84 97 22

*Klinisk ansvarlig:*Professor Pernille Tine Jensen

pernille_jensen@dadlnet.dk

Med venlig hilsen

Projektgruppen


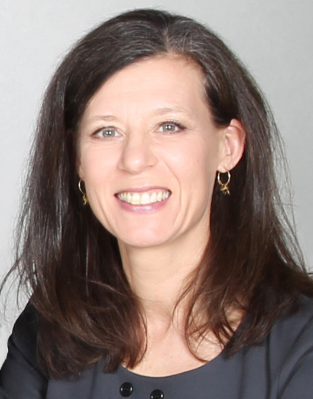


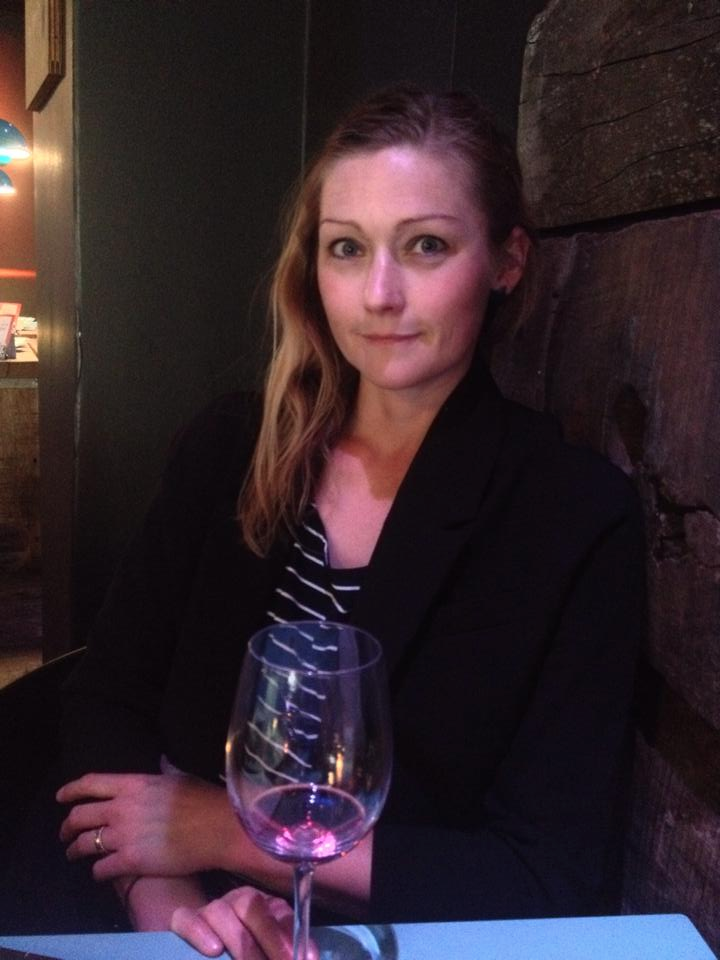


Stinne Holm Bergholdt

Læge, ph.d, post doc

Pernille Tine Jensen

Overlæge, professor


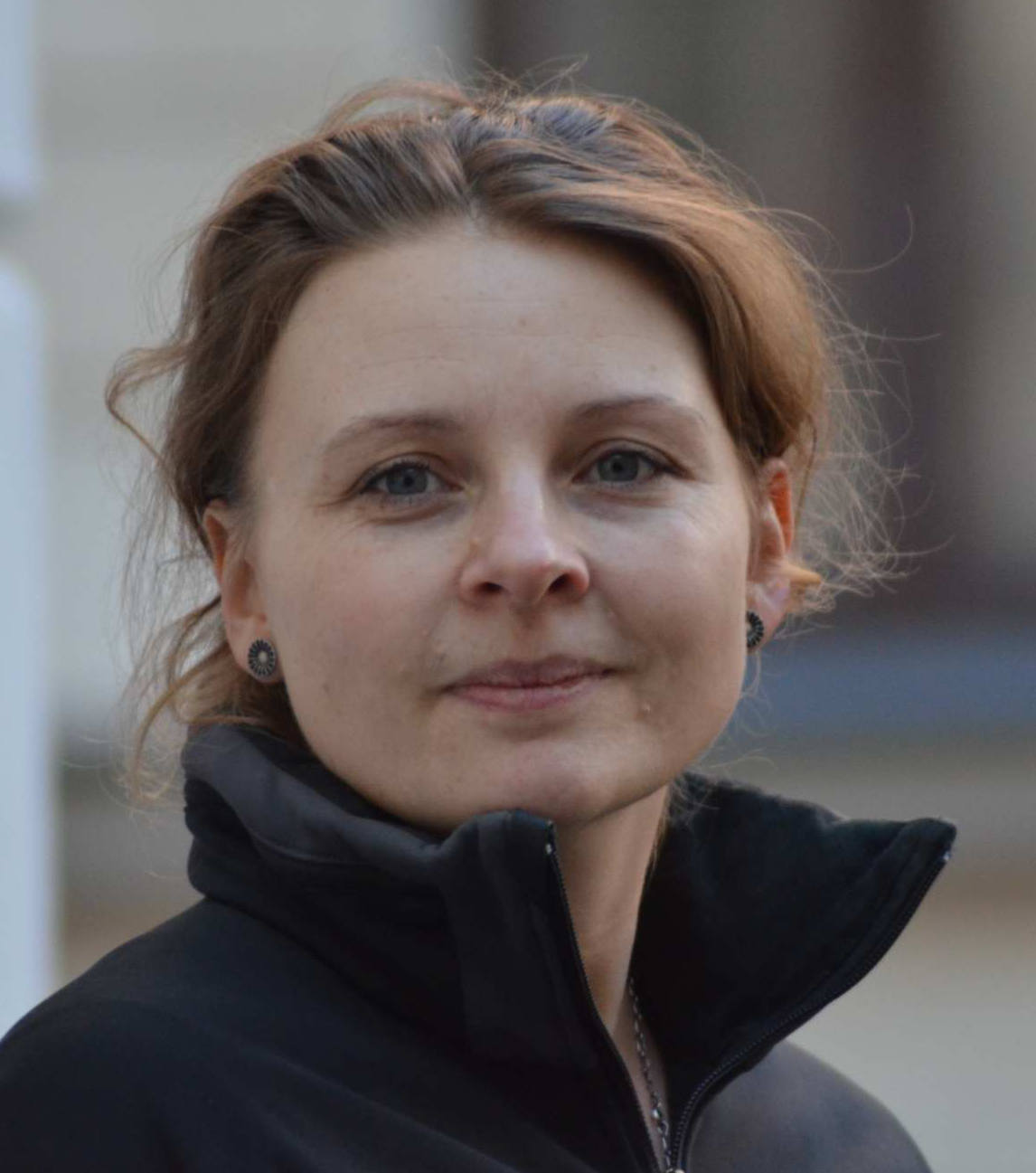


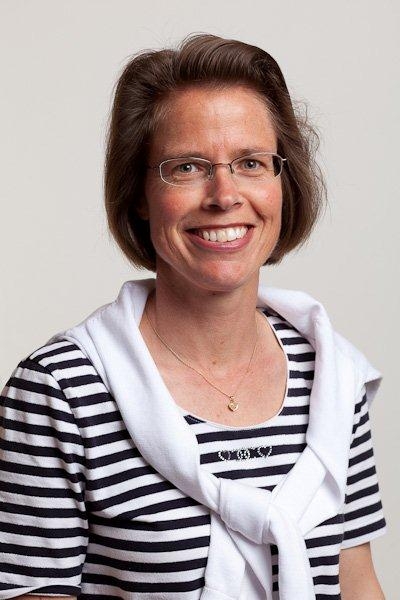


Dorte Gilså Hansen
Centerleder, lektor, læge, ph.d.

Anna Thit Johnsen

Psykolog, seniorforsker


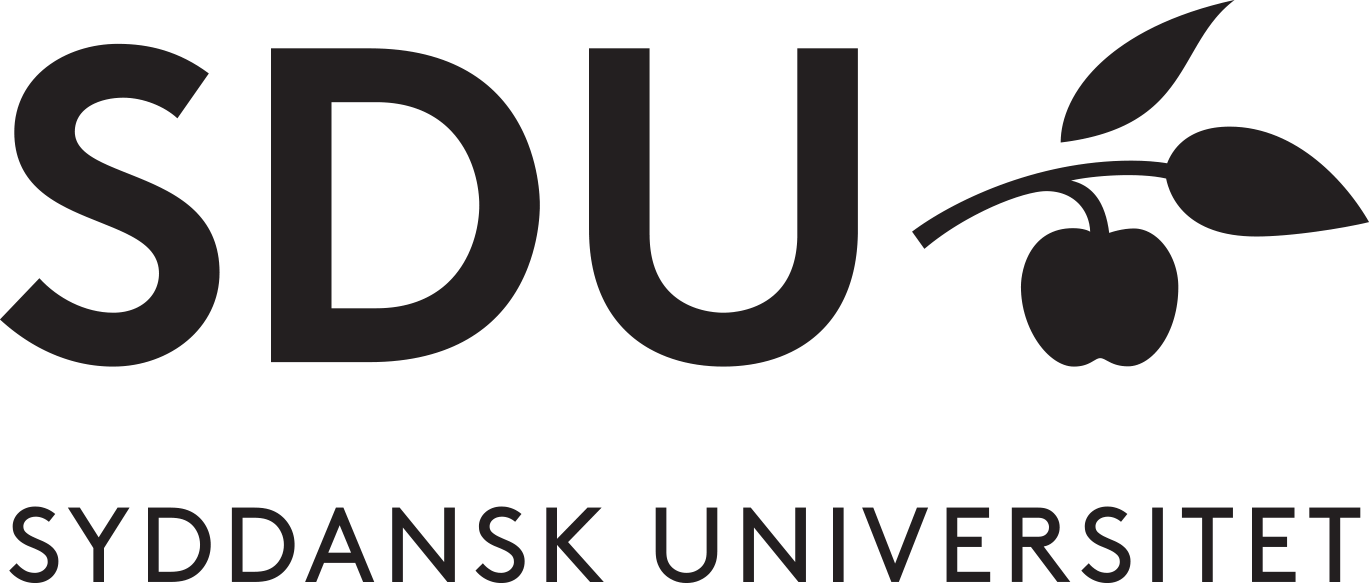


**Notater:**

**Forsøgspersoners rettigheder i et sundhedsvidenskabeligt forskningsprojekt**

Som deltager i et sundhedsvidenskabeligt forskningsprojekt skal du vide at:

• Din deltagelse i forskningsprojektet er helt frivillig og kun kan ske efter, at du har fået både skriftlig og mundtlig information om forskningsprojektet og underskrevet samtykkeerklæringen

• Du til enhver tid mundtligt, skriftligt eller ved anden klar tilkendegivelse kan trække dit samtykke til deltagelse tilbage og udtræde af forskningsprojektet. Såfremt du trækker dit samtykke tilbage påvirker dette ikke din ret til nuværende eller fremtidig behandling eller andre rettigheder, som du måtte have

• Du har ret til at tage et familiemedlem, en ven eller en bekendt med til informationssamtalen

• Du har ret til betænkningstid, før du underskriver samtykkeerklæringen

• Oplysninger om dine helbredsforhold, øvrige rent private forhold og andre fortrolige oplysninger om dig, som fremkommer i forbindelse med forskningsprojektet, er omfattet af tavshedspligt

• Opbevaring af oplysninger om dig, herunder oplysninger i dine blodprøver og væv, sker efter reglerne i lov om behandling af personoplysninger og sundhedsloven

• Der er mulighed for at få aktindsigt i forsøgsprotokoller efter offentlighedslovens bestemmelser. Det vil sige, at du kan få adgang til at se alle papirer vedrørende din deltagelse i forsøget, bortset fra de dele, som indeholder forretningshemmeligheder eller fortrolige oplysninger om andre

• Der er mulighed for at klage og få erstatning efter reglerne i lov om klage- og erstatningsadgang inden for sundhedsvæsenet. Hvis der under forsøget skulle opstå en skade kan du henvende dig til Patienterstatningen, se nærmere på www.patienterstatningen.dk
