# Supplementary material for: A NEw MOdel of individualized and patient-centered follow-up for women with gynecological cancer (the NEMO study)—protocol and rationale of a randomized clinical trial
Source: Trials. 2023 Feb 1;24:74. doi: 10.1186/s13063-022-07022-0 (PMC9890753; doi:10.1186/s13063-022-07022-0)
Supplement: Supplementary file 2 — Additional file 2. Appendix 2: Patient consent form NEMO (in Danish). [file 13063_2022_7022_MOESM2_ESM.pdf]

SAMTYKKEERKLÆRING NEMO STUDIET

Fra kontrol til individualiseret opfølgning for kvinder med gynækologisk kræft
– et randomiseret, kontrolleret studie

Patientlabel<br>(Navn og cpr-nummer)

A1: Samtykkeerklæring fra forsøgsdeltageren:

Jeg har fået skriftlig og mundtlig information, og jeg ved nok om formål, metode, fordele og ulemper til at sige ja til at deltage.

Jeg ved, at det er frivilligt at deltage, og at jeg altid og uden begrundelse kan trække mit samtykke tilbage uden at miste mine nuværende eller fremtidige rettigheder til behandling.

Jeg giver samtykke til, at deltage i forskningsprojektet, og at forskningsgruppen må indhente journaloplysninger og at mine udfyldte spørgeskemaer efterfølgende må opbevares beskyttet i dansk dataarkiv med mulighed for, at kunne anvendes i yderligere forskning på et senere tidspunkt.

Jeg har fået en kopi af dette samtykkeark samt en kopi af den skriftlige information om projektet til eget brug.

Forsøgspersonens navn: ____

Dato: _______________ Underskrift: ____

Ønsker du at blive informeret om forskningsprojektets resultat samt eventuelle konsekvenser for dig?:

Ja _____ (sæt x) Nej _____ (sæt x)

A2: Samtykke til journalindblik, hvis patienten ikke ønsker at deltage aktivt:

Jeg har fået skriftlig og mundtlig infornation om projektet og ønsker ikke at deltage aktivt i forskningsprojektet, men giver samtykke til, at forskningsgruppen må indhente journaloplysninger om min baggrund, sygdom og medicin.
Jeg ved at det er frivillingt at tillade journalindblik, og at jeg altid og uden begrundelse kan trække mit samtykke tilbage, uden at det påvirker mit behandlingsforløb.
Jeg har fået en kopi af dette samtykkeark, samt en kopi af den skriftlige information om projektet til egen brug.

Dato: _______________ Underskrift: ____

B: Erklæring fra den, der inkluderer patienten:

Jeg erklærer, at forsøgspersonen har modtaget mundtlig og skriftlig information om forsøget.
Efter min overbevisning er der givet tilstrækkelig information til, at der kan træffes beslutning om deltagelse i forsøget.

Navnet på den, der har afgivet information: ____

Dato: _______________ Underskrift: ____

Projektet er anmeldt til Datatilsynet: 16/43209 og godkendt af den lokale Videnskabsetisk Komite i Region Syddanmark: ID S-20180058. Samtykkeerklæringen returneres til projektansvarlig læge (lægges i NEMO mappen) Stinne Holm Bergholdt, mail stinne.holm.bergholdt@rsyd.dk
